# Marina crystal minerals (MCM) activate human dendritic cells to induce CD4+ and CD8+ T cell responses *in vitro*

**DOI:** 10.1177/2058738418797768

**Published:** 2018-10-01

**Authors:** Mamdooh H Ghoneum, Takeshi Ogura, James K Gimzewski, Aya D Ghoneum, Michael C Henary, Sudhanshu Agrawal

**Affiliations:** 1 Department of Surgery, Charles R. Drew University of Medicine and Science, Los Angeles, CA, USA; 2 Kaiyo Kagaku Co., Ltd., Tokyo, Japan; 3 Department of Chemistry and Biochemistry, University of California, Los Angeles (UCLA), Los Angeles, CA, USA; 4 California NanoSystems Institute (CNSI), University of California, Los Angeles (UCLA), Los Angeles, CA, USA; 5 Department of Psychology, University of California, Los Angeles (UCLA), Los Angeles, CA, USA; 6 Division of Basic and Clinical Immunology, University of California, Irvine (UCI), Irvine, CA, USA

**Keywords:** CD4+ T cells, CD8+ T cells, DCs, MCM

## Abstract

Marina crystal minerals (MCM) are a mixture that contains crystallized minerals along with trace elements extracted from seawater. It is a nutritional supplement that is capable of enhancing natural killer (NK) cell activity and increasing T and B cell proliferation in humans post ingestion. However, its effect on dendritic cells (DCs), the cells that bridge innate and adaptive immunity, is not yet known. In this study, we examine the stimulatory effects of MCM on DCs’ maturation and function in vitro. Human monocyte–derived DCs were treated with MCM at two different concentrations (10 and 20 µg/mL) for 24 h. Results showed that MCM treatment activated DCs in a dose-dependent fashion. It caused the upregulation of costimulatory molecules CD80, CD86, and HLA-DR, and prompted the production of DC cytokines, including interleukin (IL)-6, IL-10, tumor necrosis factor (TNF)-α, and IL-1β, and chemokines (monocyte chemotactic protein-1 (MCP-1)) and interferon-gamma-inducible protein-10 (IP-10). In addition, activated DCs primed CD4+ T cells to secrete significant amounts of interferon gamma (IFN-γ), and they also stimulated CD8+ T cells to express higher amounts of CD107a. These results indicate that MCM is a potentially powerful adjuvant, from natural materials, that activates human DCs in vitro and therefore may suggest its possible use in immune-based therapies against cancer and viral infections.

## Introduction

Marina crystal minerals (MCM) are a natural mixture containing crystallized minerals along with trace elements from the Oharai Sea in Japan. It is processed by condensing and reducing pure seawater to a powder through a sterilizing sequence of heating, freezing, and drying. The product contains 27 minerals and trace elements; no harmful trace elements have been detected in it.^1^ Our earlier studies showed the immunomodulatory effect of MCM to activate natural killer (NK) cells and increase T and B cell proliferation in humans post ingestion.^1^ However, its effect on dendritic cells (DCs) has not yet been discovered.

DCs, the professional antigen-presenting cells (APCs), activate adaptive immunity through their capacity to capture, process, and then present antigens to T cells.^2,3^ DCs are usually localized in non-lymphoid tissues under healthy conditions and reside in an immature state. Immature DCs are highly phagocytic for peptide uptake and processing, and they respond to signals via different receptors including scavenger receptors, nucleotide oligomerization domain (NOD)-like receptors, and toll-like receptors (TLRs). Immature DCs also respond to inflammatory mediators, chemokines, and cytokines.^4^ The conversion of immature DCs to mature DCs is associated with both phenotypic and functional changes. Maturation is characterized by the increased expression of costimulatory molecules, redistribution of HLA-DR molecules, and increased presentation of antigen and secretion of cytokines, such as interleukin (IL)-12, IL-15, and type I interferons (IFNs I).^5,6^ Mature DCs prime Th cell responses,^7^ induce the differentiation of CD8+ T cells into effector cytotoxic T lymphocyte (CTL), and have the ability to activate NK cells’ cytotoxicity.^8,9^

This study examines the ability of MCM to activate DCs with respect to phenotypic changes, including the type of cytokines secreted, and to examine the role of MCM-stimulated DCs on the activation of CD4+ T cells and CD8+ T cells, as well as the underlying mechanisms of its effect. Our results indicate that MCM is a potentially powerful adjuvant, made from natural materials, that is capable of activating DCs and therefore may be beneficial for provoking an effective immunological response against cancer and infections.

## Materials and methods

### Antibodies and reagents

The antibodies and reagents that were used in this study include CD107a (clone H4A3), CD8 PerCP (clone SK1) CD25 FITC (Clone M-A251), CD4 PerCP (Clone SK3), CD11c APC (Clone B-ly6), HLA-DR PerCP (Clone L243 (G46-6)), CD80 PE (Clone L307.4), and CD86 PE (Clone 2331 (FUN-1)). All of these were acquired from BD Biosciences (San Jose, CA, USA). For a negative control, we used an isotype antibody (BD Biosciences). *E. coli* lipopolysaccharides (LPS) were obtained from InvivoGen (San Diego, CA, USA).

### MCM

MCM is a mixture that contains crystallized minerals along with trace elements and other active ingredients, extracted from seawater and originally separated from sodium chloride. MCM was prepared for use by dissolving in complete medium (CM), resulting in a range of concentrations (10 and 20 μg/mL). We received MCM for this study from the Foundation for Basic Research Institute of Oncology, Japan (MCM was provided by Kaiyo Kagaku Co., Ltd, 3-11-5 Minami Azabu, Minato-ku, Tokyo 106-0047, Japan).

### CM

CM consists of RPMI 1640 supplemented with 10% fetal bovine serum (FBS), 1 mM glutamine, 100 U/mL penicillin, and 100 µg/mL streptomycin.

### Isolation and culture of human monocyte–derived dendritic cells

We prepared monocyte-derived dendritic cells (moDCs) for this study as described previously.^10^ In summary, peripheral blood mononuclear cells (PBMCs) from heparinized blood, obtained from donors who were normal and healthy (approved by the Institutional Review Board (IRB), Charles Drew University), were separated using Ficoll-Hypaque density gradient centrifugation. We then allowed the cells to attach to culture plates for 2 h. Any cells not adhering to plates were removed. Monocytes adhering to plates were then cultured for 6 days within a humidified atmosphere, containing 5% CO_2_ at 37°C in RPMI 1640 supplemented with 10% FBS, 1 mM glutamine, 100 U/mL penicillin, 100 μg/mL streptomycin, human granulocyte-macrophage colony stimulating factor (GM-CSF) at 50 ng/mL (PeproTech, Rocky Hill, NJ, USA), and 10 ng/mL recombinant human IL-4 (PeproTech). We discarded half of the culturing medium every 2 days and replaced it with fresh medium. After 6 days, DCs were collected, and we measured the purity of the obtained DCs to be >95%. We then pulsed DCs with either 1 μg/mL *E. coli* LPS, used as a positive control, or MCM (10 and 20 μg/mL) for 24 h.

### DC phenotyping

We determined the expression of cell surface markers by employing flow cytometry. FACS analysis-flow cytometry was performed using FACSCalibur (Becton-Dickenson, San Jose, CA, USA) and analyzed using FlowJo software (Tree Star, Ashland, OR, USA). In summary, we analyzed gated CD11c+ HLA-DR+ DCs for the expression of CD80, CD86, and HLA-DR. We received the appropriate antibodies from BD Pharmingen (San Diego, CA, USA). Viability of DCs was tested by trypan blue; more than 95% cells were live.

### Cytokine production by DCs

MoDCs were incubated with MCM at the concentrations of 10 and 20 μg/mL for 24 h. We collected supernatants and stored them at −70°C until analysis. We measured the cytokines IL-6, IL-10, tumor necrosis factor (TNF)-α, IL-1β, monocyte chemotactic protein-1 (MCP-1), and interferon-gamma-inducible protein-10 (IP-10) (BD Pharmingen) in the supernatants using specific enzyme-linked immunosorbent assay (ELISA) kits, following the manufacturer’s protocol.

### DC-CD4+ T cells

We purified allogenic CD4+ T cells by negative selection by employing a magnetic bead–based kit, which we acquired from Stem Cell Technologies (Vancouver, BC, Canada). We then cultured allogenic CD4+ T cells with DCs that had been stimulated with MCM (10 and 20 μg/mL) for 24 h as described above. We co-cultured the DC-CD4+ T cells for a total of 5 days in a U-bottom 96-well plate. The DC:CD4+ T cell ratio was 1:5 (2 × 10^4^:1 × 10^5^). After 5 days, the supernatants were collected and kept at −70°C. We subsequently detected the cytokines IFN-γ, IL-10, and TNF-α by employing a specific ELISA kit (BD Pharmingen) IL-22 (R&D systems, Minneapolis, MN, USA). Viability of cells was tested by trypan blue; more than 95% cells were live.

### DC + T cells

We enriched allogenic T cells by negative selection by employing a magnetic bead–based kit, which we acquired from Stem Cell Technologies. We cultured MCM-stimulated DCs with T cells in 96-well plates, with the ratio of DCs to T cells of 1:5. After 5 days, supernatants were collected and cells were stained for the surface markers CD4, CD8, CD107a, and CD25. Viability of cells was tested by trypan blue; more than 95% cells were live.

### Statistics

In this study, we repeated all of the experiments with samples from 5–7 individual subjects. We tested the probability of the mean values of two experimental groups by the two-tailed t-test for paired samples. We set the level of significance at *P* < 0.05. We performed statistical analysis for bar graphs by employing GraphPad Prism software.

## Results

### MCM activates DCs and upregulates costimulatory molecules

**Figure 1.**
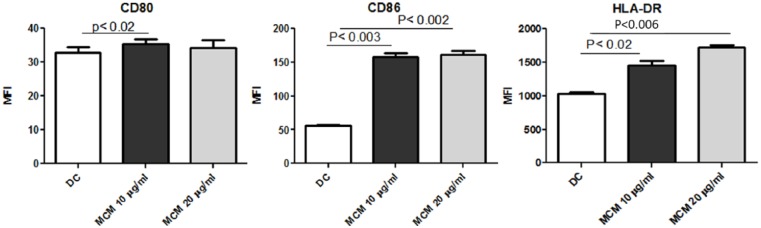
Upregulation of costimulatory and maturation molecules CD80, CD86, and HLA-DR on MCM-treated DCs. The mean florescence intensity (MFI) of CD80, CD86, and HLA-DR in DCs post treatment with MCM. Data represent the mean ± SE of three experiments. Values are considered significant at *P* < 0.05 as compared to DCs alone.

MoDCs (1 × 10^6^/mL) were cultured with MCM for 24 h. Flow cytometry was used to measure the expression and density of maturation markers. Figure 1 shows the mean fluorescent intensity (MFI) of CD80, CD86, and HLA-DR in DCs. MCM treatment caused a dose-dependent increase in the expression of DC surface costimulatory and maturation markers CD80, CD86, and HLA-DR. This increase was detected at a concentration of 10 µg/mL and further increased at 20 μg/mL. In comparison with untreated DCs, it can be seen that treatment with MCM significantly upregulates the expression of CD80, CD86, and HLA-DR markers.

### MCM induces cytokine production by moDCs

**Figure 2.**
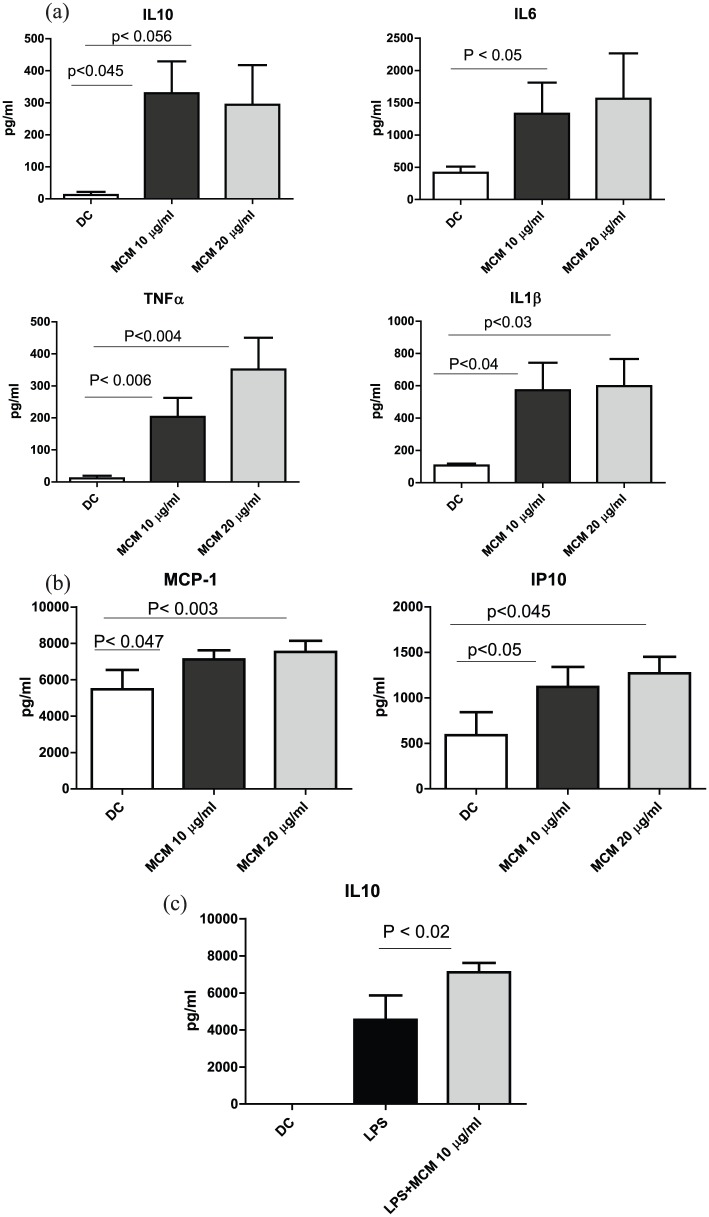
MCM activates DCs to secrete cytokines and chemotactic proteins, and enhances IL-10 secretion on LPS stimulation. (a) Cytokine production by MCM-treated moDCs. Results are expressed as mean ± SE from seven individual experiments. (b) Secretion of chemotactic proteins MCP-1 and IP-10. Data represent the mean ± SE of five experiments. (c) MCM enhances IL-10 secretion on LPS stimulation. Data represent the mean ± SE of seven experiments. Values are considered significant at *P* < 0.05 as compared to DCs alone.

MCM at the concentrations of 10 and 20 μg/mL appear to be non-toxic to the normal cells (human moDCs). Data in Figure 2(a) show that MCM has the ability to activate DCs to induce cytokine production, such as IL-6, IL-10, TNF-α, and IL-1β. The levels of cytokine secretions post treatment with MCM was compared with moDCs alone. IL-6 production was increased by about threefold at the concentrations of 10 and 20 μg/mL. MCM at a low concentration of 10 µg/mL also induced a significant increase in IL-10 production. The level of activation did not increase with increasing the concentration to 20 μg/mL. In addition, MCM was able to activate TNF-α production in a dose-dependent manner. Furthermore, IL-1β production was significantly increased by sixfold at the concentrations of 10 and 20 µg/mL.

### MCM induces chemokines secretion by DCs

Chemotactic proteins MCP-1 and IP-10 are known to help DCs migrate to the lymph nodes. The levels of MCP-1 and IP-10 were examined post treatment of DCs with MCM. Results in Figure 2(b) show that treatment with MCM caused a twofold increase in the level of IP-10 at a low concentration (10 μg/mL) and further increased at a higher dose of MCM (20 μg/mL). MCM can also activate MCP-1 in a dose-dependent manner.

### MCM enhances IL-10 secretion on LPS stimulation

Data in Figure 2(c) show that DCs were stimulated with LPS alone and LPS + MCM. A siginificantly higher secretion of IL-10 was observed; however, there was no change in the levels of TNF-α, IL-6, IP-10, and MCP-1 (data not shown).

### MCM-stimulated DCs prime CD4+ T cells and secrete significant amounts of IFN-γ and IL-22

**Figure 3.**
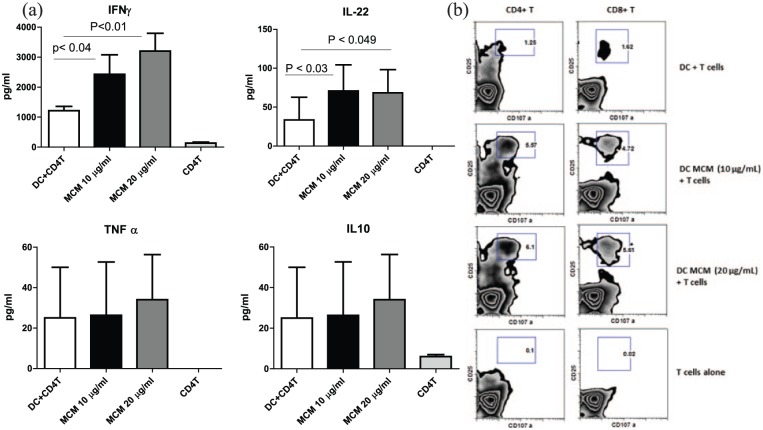
(a) MCM-stimulated DCs prime CD4+ T cells to secrete IFN-γ, IL-10, IL-22, and TNF-α. Data represent the mean ± SE from five individual experiments; values are considered significant at *P* < 0.05 as compared to DC-CD4+ T cells alone. (b) MCM-stimulated DCs activate CD4+ and CD8+ T cells to express higher amounts of CD25 and CD107a. One representative experiment is shown from five individual experiments.

Figure 3(a) shows the secretion levels of IFN-γ, IL-10, IL-22, and TNF-α. Results show that IFN-γ levels were significantly increased post treatment with MCM as compared to DC-CD4+ T cells alone (*P* < 0.05). We observed an increase in the cytokine levels of IFN-γ by three-to fourfold and IL-22 also by twofold. However, MCM-treated DCs did not induce the secretion of IL-10 and TNF-α over unstimulated controls.

### MCM-stimulated DCs activate CD4+ and CD8+ T cells upregulating a higher amount CD25 and CD107a

Data in Figure 3(b) show that MCM-treated DCs activate CD4/CD8 cells and upregulate CD25 expression which is a marker of activation. These CD25+ cells also display upregulated expression of CD107a which is a marker of degranulation expressed in cytotoxic T Cells.

## Discussion

DC maturation and activation is an essential step for DCs to mount effective immune responses against infections and for cancer immunotherapy. In this study, MCM, a crystallized mixture of 27 minerals and trace elements from seawater, was shown to be a potent activator of human DC maturation and function. MCM-activated DCs induced CD4+ and CD8+ T cell responses in vitro as manifested by CD4+ T cell production of the cytokines IFN-γ and IL-22 and higher CD107a expression in both types of T cells. In addition, MCM-activated DCs caused a dose-dependent upregulation of costimulatory and maturation marker expressions on the surface of DCs including CD80, CD86, and HLA-DR. MCM at 10 μg/mL markedly increased DCs’ cytokine secretion (IL-6, IL-10, TNF-α, and IL-1β), chemokine secretion (MCP-1 and IP-10), and IL-10 secretion on LPS-activated DCs.

While the mechanisms underlying MCM’s activation of DCs are not fully understood, they might be due to the ability of MCM to bind to receptors on the DC surface, subsequently triggering the signaling pathways involved in DC activation. Alternatively, signal cell activation pathways could be achieved through possible binding of MCM to intracellular receptors such as NLRP3 inflammasome since it secretes IL-1β. Any of MCM’s several minerals and trace elements might contribute to the activation of DCs. Given the range of literature linking zinc, magnesium, copper, and iron with immunological responses, we tentatively favor the presence of these elements in MCM as primary contributors to MCM’s induction of phenotypic and functional changes in DCs.

Previously, MCM has been shown to exert an apoptotic effect against human LNCaP prostate cancer cells in vitro^11^ and to activate NK cells in humans post ingestion.^1^ In our study, MCM enhanced the cytotoxic effect of DC-CD8+ T cells and stimulated DCs to prime CD4+ T cells and secrete significant amounts of IFN-γ, all of which are known to exert antitumor activity.^12^ Taken together, these results suggest that MCM exerts its anti-cancer activity by mounting different arms of the immune system.

This study indicates that MCM is a potent natural dietary adjuvant that effectively activates human DCs and suggests MCM’s potential use against cancer and viral infections via DC-based vaccine strategies in multiple clinical trials.
